# Using Oral Rehydration Therapy (ORT) in the Community

**DOI:** 10.3390/tropicalmed6020092

**Published:** 2021-05-29

**Authors:** Richard A. Cash

**Affiliations:** Department of Global Health and Population, Harvard T.H. Chan School of Public Health, Boston, MA 02115, USA; racash@hsph.harvard.edu

**Keywords:** oral rehydration solution (ORS), oral rehydration therapy (ORT), community-based care, OTEP, BRAC

## Abstract

For ORT to have a maximum impact on public health it should be used in the community, in the home. A number of programs have been developed over the years to extend ORT to home use. One of the most successful approaches was the Oral Therapy Education Program (OTEP) developed by BRAC, the world’s largest NGO. Mothers were taught in the home by an OTEP worker using seven simple messages and a demonstration. The program, which led to high levels of use and knowledge retention, is described. What the OTEP and other successful home-based programs have demonstrated is that home care of diarrhea using ORS can be effectively implemented and can have a positive impact on the reduction of diarrhea morbidity and mortality.

## 1. Introduction

The development and proof-of-concept of oral rehydration solution (ORS) and the treatment package of oral rehydration therapy (ORT) were many years in the making, from physiologic studies to the first clinical applications in 1968 [1]. The reasoning behind this effort was to expand therapy to those areas where intravenous (IV) fluid, needles, and IV tubing were not available and where health personnel were in very short supply—that is, in most countries where cholera was a significant health problem. ORS was to be an intervention for use not only in health facilities but also in the home. Though ORS was originally developed for cholera, there is increasing evidence that it is effective in all types of infectious diarrheas, ranging from rotavirus to the recent demonstrations of its effectiveness in Ebola-related diarrhea. It seems to be a universal treatment.

For ORS to be most effective in reducing diarrhea morbidity it has to be used where cases occur. This article examines efforts to extend the use of ORS to the community—to local practitioners and mothers and other home-based care givers. 

## 2. Early Efforts

Soon after the clinical studies demonstrated the effectiveness of ORS in treating adult and childhood cholera and non-cholera diarrhea, efforts began to extend care to rural treatment centers and the home. Early interventions focused on producing packets of electrolytes and glucose for wide distribution. Packets were originally designed to be added to one liter of potable water, with instructions written on the packet. The World Health Organization (WHO) heavily endorsed this concept, and training courses were set up worldwide to teach doctors how to use ORS. The United Nations Children’s Fund (UNICEF), under the leadership of Jim Grant, established a number of facilities for the production of ORS packets. The United States Agency for International Development (USAID) was a major contributor to conferences and workshops to help disseminate knowledge about ORS and to support programs to expand distribution. Packets were an attractive idea as they standardized the composition of ORS, were easy to distribute, and could be sold by shops and pharmacies. There were flaws in this strategy, however, especially in the poorest countries. A mother or other child caregiver had to be literate to read the instructions; the person making up the solution had to have a liter container to mix the water and salts; and there had to be an effective distribution system to send the packets to the village and home. Despite these issues, WHO was initially opposed to any other methods of delivery and any change in the formula. One size was designed to fit all, including the size of the packet. 

Use of ORS in the community was less extensive than might have been expected both because of these limitations and the resistance of some health providers, especially doctors. To many it seemed counterintuitive to give oral fluids when the child had diarrhea, and there was little money to be made in selling such an inexpensive product. This led a number of governments and community-based organizations to try other methods of delivering ORS and increasing use. Rather than attempting to review these many efforts to increase community and home-based use, this article will focus on one program that used innovative strategies to greatly increase use in a very large population—the Oral Therapy Education Program (OTEP) of BRAC, a large Bangladeshi non-governmental organization (NGO) [2]. 

## 3. The BRAC OTEP Program

Founded in 1972, BRAC is a Bangladeshi NGO that is presently the largest in the world and has been ranked the number-one NGO globally over the past 5 years. BRAC is especially known for scaling up programs to reach millions, whether it be through microcredit, mobile banking, primary education, water and sanitation, health interventions, or multiple other development programs. In 1980 OTEP began a 10-year effort to educate every Bangladeshi mother about ORS and to teach them how to prepare and use the solution. 

As in so many low-income countries, diarrhea was the cause of up to 30% of infant and child deaths. There was a significant shortage of MBBS physicians (the degree of a trained allopathic doctor), estimated at less than 1 per 10,000 in rural areas, and fewer nurses, with most care delivered by unlicensed village doctors (or village “quacks”) or through local drug sellers in village shops. Based on the WHO approach, the government established the National Oral Rehydration Program (NORP) which was designed to distribute the oral therapy packets to pharmacies and treatment facilities, and to teach doctors throughout the country. The number of treatment facilities was limited and most did not have a full complement of health personnel. Female literacy was less than 5% and there were no standard 1-liter containers in the countryside. It was no wonder that knowledge and use with respect to ORS were limited. BRAC concluded that the only means of spreading the use of ORS to reduce diarrhea morbidity and mortality was to teach women, all women, how to prepare and use ORS in the village, in the home. 

Treatment was literally put in the hands of the mother. A local experiment indicated that a 3-finger pinch of salt (labon) and a hand scoop of raw sugar (gur, which is half glucose and half fructose but also contains potassium) dissolved in a half a liter of water (500 cc) produced an oral solution with a sodium content of approximately 50 mEq/liter. This labon-gur solution (LGS) was tested in adult patients with non-cholera diarrhea treated at the Cholera Research Laboratory in Dhaka (now the icddr, b). The outcome was similar to that of a group with mild to moderate diarrhea given the standard WHO-recommended solution. Though WHO had serious reservations about promoting incomplete home-based solutions, BRAC began field tests, convinced that successful treatment of child diarrhea lay in convincing the mother to hydrate with an available and appropriate solution, and that the packet-based program would not work in rural Bangladesh. 

Though a national program was not yet being considered by BRAC there were four questions that remained to be answered in designing their program: What should be the message content? Who should be the recipients of the message? What should be the method of teaching? Who should be the teachers? The response to each of these questions would shape the creation of the program and how it was to be evaluated.

Mothers were the obvious recipients of the message, and given that less than 5% were literate, the message had to be crafted so that literacy was not required. To reach mothers in the village, teaching had to be provided in the home by women. Lastly, the message had to be simple and practical, with a primary focus on teaching how to prepare the solution. Originally, in the program it was decided that the message would involve 17 points. The message was reduced to 10, and finally 7 essential points (see Box 1) [2]. What is not often appreciated is that it takes much more skill to simplify than to complicate. This is often true of public health messaging, which often suffers from providing unnecessary information in a long format.

Box 1 **The 7 points to remember in the BRAC OTEP program were as follows.**

1. Defining different terms for diarrhea—i.e., “dud haga”, “ajirno”, “amasha”, “daeria”, or “cholera” and their effects

2. Symptoms of diarrhea

3. Simple management of loose motions (replacing salt and water in the body)

4. Preparing ORS (3-finger pinch of salt, 1 fistful of gur/sugar, dissolved in 1 half-seer of water or 500 cc

5. Administration of ORS

6. Advice on nutrition (continued breastfeeding, rice, curry, other household foods)

7. Prevention (drinking water from a safe source, keeping food clean, breastfeeding)

Teachers were called oral replacement workers (ORW). The ORWs were all women recruited from the districts in which the program was conducted. They were 20–35 years of age, had about 10 years of schooling, and did not have children younger than 3 years of age. The original training was for 5 days and focused on teaching techniques and recordkeeping. There were daily feedback meetings and a 1-day refresher course every 3 months. 

Each ORW visited up to 10 women a day with each encounter lasting about 20 min. All the ORWs were recruited from the villages. The ORW would go house to house, teaching the method of a 3-finger pinch of salt (labon) and a fistful of raw sugar (gur) or refined sugar to each half-liter of water. This will be referred to as the “labon-gur” solution. To ensure the mother had a half-liter container, the ORW carried the proper-sized cup. She requested the mother to bring any container from the kitchen into which a half a litter of water was poured and a scratch mark made on the pot to designate how much water should be used. The water used was the best available drinking water normally used in the home. Boiling the water was not encouraged as it added another step, increased fuel consumption, and there were no studies indicating that childhood diarrhea would worsen if contaminated water was used. 

The ORWs were not given a fixed salary but were paid on the basis of their performance as educators. That is, the more the mothers learned, the more they were paid. To do this it was necessary to develop an effective monitoring system. Each ORW kept a list of the mothers she taught. A supervisor would randomly select a 10% sample that would be given to the monitor to visit and interview. All monitors were young men who could travel from village to village, something that a single woman could not do. The monitors had no direct contact with the ORWs. 

The mother was asked about the 7 points and to prepare the labon-gur solution. Her responses were graded according to the following criteria: A—answered 6–7 questions and prepared the solution; B—answered 4–5 questions and prepared the solutions; C—answered 2–3 points and prepared the solution; D—could not prepare the solution. From these results the ORW salary was calculated. For all mothers graded as A, the worker received Taka (Tk) 4 (USD 0.025 using the 1980 exchange rate) multiplied by the number of mothers she had seen in a given period of time; for B she received Tk 2; for C she received Tk 1; and for those graded D she received no compensation. Major emphasis was placed on ensuring that the mother knew how to correctly prepare the labon-gur solution. 

If the program maximized worker payments it was because mothers were learning. The incentive system resulted in more attention being given to improving the teaching technique, and suggestions came from the bottom up. As the ORW demonstrated and then asked the mothers to prepare the solution, communication with the mother increased, as did interest in the teaching session. 

## 4. Scaling Up the OTEP 

The mantra of the BRAC founder, F.H. Abed, was “Small is beautiful but big is necessary”.

When the pilot program of OTEP demonstrated that ORS could be prepared by village women who remembered the 7 points, the next step was to plan to teach every mother in the country in order to scale up the program. Small groups of 6–10 ORWs fanned out across the country, spending 4 weeks at a community facility at the union level (a union is the smallest administrative unit in Bangladesh and is composed of 9 villages). They were accompanied by a cook and a chowkidar (watchman). From the community center they reached all the villages in a union. As the program expanded it continued to evolve based on feedback from the OTEP staff, the Technical Advisory Committee, and the Research and Evaluation Division of BRAC. Some important changes included: establishing field labs to monitor the quality of the ORS; revising the 7 points; involving men at village markets; teaching about ORS in schools; increasing the length of stay in a union from 4 to 6 weeks; teaching small groups rather than individuals; improved monitoring; and experimenting with cereal-based ORS. As men were often the decision makers deciding what treatment should be given to the child, they also had to be informed of the method and its value.

Factors affecting the successful scaling-up of OTEP can be summarized as follows: management; performance-linked salary; planning; recruitment; training and staff development; communication; logistics; feedback and coordination; staff commitment; government support; international support; use of outside expertise; and funding. 

Was the OTEP a success? As the objective of the OTEP was to teach each and every mother in Bangladesh to prepare and use ORS to treat diarrhea, success could be measured by different criteria: coverage; knowledge; management; use, impact; replication; and sustainability. In the evaluation of any intervention, time is a critical factor. How long should it take to change ideas and practice? The OTEP program lasted 10 years, so it should be evaluated over that period of time. OTEP field workers visited all the villages (except a few in tribal districts where there was civil unrest), which translated into 12 million households, though the number of women taught was higher as some households had more than 1 woman. Over 90% of mothers knew of and could prepare ORS, and the vast majority of them were capable of preparing a clinically safe and effective solution. Even 15 years after the initial encounter with an OTEP worker, ~70%, reported knowledge and use. 

An important indicator of success is how frequently and the degree to which ORT is used in treating an episode of diarrhea. If all types of diarrheas—mild, moderate, or severe, watery or non-watery—were included in the denominator, about half of the episodes were treated with ORT (LGS, packet ORS, or other rehydrating fluids). If the denominator was more rigidly defined as only more severe cases, use increased to 82%. Today, Bangladesh has the highest use rate of ORT to treat diarrhea of any country. 

Measuring mortality from a constellation of conditions like diarrhea is problematic as there are many causes and contributing factors to overall mortality, especially in environments where mortality is high. Studies examining “process” measures (retention of knowledge, use rates, perception, etc.) have been far more useful and much less costly. If mothers retain the knowledge, they are more likely to use ORT with increased frequency and volume if diarrhea appears to be more severe. Evidence suggests that in higher-risk groups (infants, children, and the elderly), diarrhea-related mortality was reduced and there’s no doubt that ORT use has contributed to lowering overall diarrhea mortality [3,4].

There are many lessons from the BRAC experience with ORT in developing the message, spreading the message, increasing use, and scaling up health education projects. One observation is that mothers, regardless of their degree of literacy, have the capacity to learn given the right kind of teaching. The message was based on previous experiences (childcare and cooking), addressed the well-recognized problem of child diarrhea, and was culturally acceptable. By preparing the solution at home using ingredients usually available, no money had to be spent by the mother unless she had to purchase gur or sugar. Simplifying the message was essential; a guiding principle of health education should be to keep the necessary ideas and discard the others. Simplifying is not “dumbing down”. 

In spreading the message in the community, different strategies were used. Firstly, the quality of teaching was linked to the salary of the ORW. This led to suggestions coming from the “bottom up” as there was a strong incentive for workers to improve their performance. The more the mothers learned, the higher the salary. The OTEP messages were reinforced through education of the men, school programs, and the use of newspapers, radio, and TV, though the latter was not widely available in rural areas. Programs were designed to reach local health care providers, with most being in the informal sector, and drug sellers. Even though there was limited monetary gain associated with increased home use of ORT, the outreach to health providers reinforced the messages, limited negative pushback, and increased their skill set. Packets of ORS salts in Bangladesh are now designed to be dissolved in a half-liter of water rather than the standard liter packet, demonstrating the degree to which the message has been adopted.

Knowledge led to increased use, which was measured in various ways but most importantly by first asking women. The use of ORT can also be deduced by the numbers of children coming to hospital with diarrhea and the state of hydration of these children. The number of visits should be reduced and the level of dehydration should improve. Measuring mortality is often at the request of a donor, but process measurements proved to be far more important. 

What are the lessons regarding the scaling up of programs such as OTEP? Observations from BRAC have been framed as follows [5]:

Innovation is a process of iteration, learning, evaluation, and implementation, requiring patience;

We should start by learning and recognize that social innovation is a constant adjustment;

We should focus on what needs to be reduced, not added—simple solutions scale easier;

Get decision makers to pay attention;

The goal is improvement, not total change.

BRAC has a clear institutional vision—the alleviation of poverty—and a commitment to scaling up. Pilot programs were developed with robust monitoring, evaluation, and research as critical components. Scaling up was achieved through a simple message and information was delivered directly. Staff were trained prior to scaling up. As a learning institution, BRAC embraced feedback and failure. Like any implementation program there were tensions within the strategy, but the program was flexible. BRAC also worked with the government, not in competition, so programs complimented each other. 

A number of global initiatives were taking place when the OTEP program began, which lent credibility to their effort and others to increase community use (see Box 2). That a simple solution could have such an impact on infant and child health and that this technology could be put in the hands of mothers changed the meaning of primary health care.

Box 2 **Policies and programs that contributed to increasing community use of ORT.**

1. Alma Ata and ”Health for All”;

2. WHO’s commitment to expanding the use of ORS;

3. UNICEF and its Growth monitoring, Oral therapy, Breast feeding and immunization (GOBI) program;

4. The role of NGOs (such as BRAC), other NGOs, and GNGOs;

5. Global meetings (e.g., ICORT I, II, III);

6. Inter-sectoral collaboration and increased support for applied research; and

7. Private sector marketing and distribution of ORS.

There were many other country programs developing their own strategy to spread the use and message of ORT. As the distribution of packets and the availability of standard containers became more widespread there was probably less need for the “pinch and scoop” method of the labon-gur approach. Packets do provide a more complete solution, with both bicarbonate and potassium in the mix. In addition to packets, some programs have focused on using cereals as a substitute for glucose (gur or sugar). Rice has been a favorite. BRAC recognized that the price of even gur was often beyond the means of the very poor and when rice/rice powder was found to be effective [6] (more so in cholera and non-vibrio cholera) and more universally available than sugar or gur, BRAC began to recommend rice for ORT [7]. As fuel must be used to cook the rice, this proved to be a disincentive for some mothers, and many preferred to use sugar. The basic message of using water and electrolytes with an absorbable substrate (e.g., glucose, sucrose, rice) to prevent dehydration, overcome deficit, and replace losses from diarrhea remained the same for all methods and has persisted. Hydrate, hydrate, hydrate. That is the important message that programs must deliver to the community. 

A final thought by Albert Einstein might summarize the BRAC experience; “Everything should be as simple as possible but not one bit simpler.” 

## Abbreviations

ORS	Oral rehydration solution
ORT	Oral rehydration therapy, which is based on the proper use of ORS and includes nutrition advice during diarrhea
OTEP	Oral Therapy Education Programme (of BRAC)
BRAC	A Bangladeshi Non-Government Organization (NGO)
UNICEF	United Nations Children’s Fund
icddr,b	International Centre for Diarrhea Disease Research, Bangladesh
